# Farmer typology and drivers of agricultural mechanization use in Haiti

**DOI:** 10.1038/s41598-024-62883-6

**Published:** 2024-05-25

**Authors:** Bénédique Paul, Jude Régis

**Affiliations:** 1 Centre Haïtien d’Innovations en Biotechnologies pour une Agriculture Soutenable (CHIBAS), Université Quisqueya, 218 Avenue Jean Paul II, HT6113 Port-au-Prince, Haiti; 2 Groupe de Recherche et d’Action pour le Développement Économique et Social (GRADES), Quartier Morin, Haiti

**Keywords:** Climate sciences, Environmental social sciences, Energy science and technology

## Abstract

Agricultural mechanization is recognized as an important technology to increase agricultural productivity, face labor shortages, and reduce post-harvest loss. However, variations among farms’ characteristics and agricultural production systems suggest adopting a targeted strategy in mechanization programs for farmers. This research aimed to answer the following questions in the particular case of Haiti: are there different types of smallholder farmers in terms of mechanization use and socio-economic characteristics? What types of mechanization are used by farmers, and what drives their use among different types of farmers? What are the different types of farms in terms of mechanization use? We used typology construction methodology (principal component analysis (PCA) and hierarchical cluster analysis (HCA)) for a sample of 637 farmers and have identified four different clusters of farmers according to the characteristics of the farms they managed: “Little rain-fed farms” (cluster 1), “Little lowlands farms” (cluster 2), “Medium-sized farms in irrigated plains” (cluster 3), and “Large fragmented mountain farms”. Farms in cluster 3 were those who used more agricultural mechanization, and the results of multinomial logistic regression (MNLR) model revealed that the significant drivers of this use were location, access to credit and low food security status. Mechanization use of farms in clusters 1 and 4 was distinctively driven by saving behavior and off-farm income, respectively. In the pooled sample, the drivers of mechanization were: regions or location, age of the farmers, irrigation, livestock, access to credit, off-farm income and food security status. This study contributes to the literature by testing new drivers of agricultural mechanization such as food security status, and off-farm income. The findings can be used to design appropriate mechanization strategies to increase productivity and face labor price/scarcity challenges. They suggest that mechanization policies should focus on agricultural equipment that are adapted to the specificities of the production systems of each farm type, and strengthen access to credit. Otherwise, mechanization will be predominantly used only in irrigated lowlands.

## Introduction

Agricultural mechanization is an important strategy to reverse the vicious circle of labor shortage—low production—food insecurity—and emigration^1,2^. It is defined as the process of improving farm labor productivity through the application of agricultural tools, implements and machinery^3^. Researchers have argued it is a key to food security in developing countries^4,5^. But, in many developing countries, farms’ constraints such as small size and low access to credit limit the successful application of improved technologies like animal and mechanical traction, labor-saving harvesting, and post-harvest processing devices, etc.^1^. Food security researchers support that lack of appropriate technology is linked to low productivity and can affect food security^6,7^, particularly in regions already threatened by land degradation and climate change. Yet, targeted technologies are needed to increase local food production and contribute to achieving the first sustainable development goal (SDG1) of zero hunger as defined by the United Nations^8^.

Agricultural development policies cannot count on a one-size-fits-all mechanization strategy^5^. In many developing countries, agriculture is dominated by small-scale farming practiced in mostly highlands^9^. Only sustainable mechanization rather than traditional one (using heavy machines) can meet the needs of farmers who use land of high declivity without accelerating soil degradation^9,10^. The Food and Agriculture Organization (FAO) has defined sustainable agricultural mechanization as all farming and processing technologies, from simple and basic hand tools to more sophisticated and motorized equipment^11^.

Haiti is a particular case in terms of agricultural mechanization. Its agriculture is mainly practiced in the highland, by more than one (1) million of fragmented farms of less than 1 ha^12^. The plains represent less than 20% of the country's surface area^2^. However, agriculture has been the most important economic occupation historically. In recent decades, the country has experienced massification of education^13^ and expansion of the tertiary sector^14^. Agricultural production declined sharply, partly because of labor shortage and labor price competition issues^2^. As a result, 4.9 out of the 12 million Haitians were facing food insecurity in 2022^15,16^.

Public agricultural policy was needed to address the causes of low agricultural productivity. Unfortunately, most interventions focused on improved varieties and intensification practices. Episodic efforts in mechanization were limited to heavy mechanical traction (tractorization) with few results in terms of sustainable use^17^. Very few Haitian farmers have adopted animal traction; most farms remain dependent on manual tools for production, harvest, and post-harvest hardship activities. In addition to persistent low productivity^18^, they usually lose around 30% of their post-harvest products each year^19^.

Since 2018, the Haitian government has initiated a turning point in its intervention approach to target smallholder farmers in different agro-ecological zones. The Ministry of Agriculture, Natural Resources and Rural Development (MARNDR) has been implementing a Program for Technological Innovation in Agriculture and Agroforestry (PITAG), with co-funding from the Interamerican Development Bank (IDB), the Global Agricultural and Food Security Program (GAFSP), and International Fund for Agricultural Development (IFAD). Among the technologies to be co-developed with farmers and disseminated among them, technical packages relating to small-scale agricultural mechanization were prioritized.

According to the literature^5^, to be appropriate and adopted, the expected mechanical innovations must take into account the diversity of Haitian farms. To this end, the following research questions were investigated: Are there different types of smallholder farmers in terms of mechanization use and socio-economic characteristics? What types of mechanization are used by farmers and what drives their use among different types of farmers?

To our knowledge, this is the first research on this topic focusing on farmers' typology about their use of mechanization in Haiti. In this country, agricultural mechanization has been limited to farmers with irrigated lowlands while most of the agricultural production used in the local food systems is realized in highlands where rain-fed crops prevail^20^. Neglected by public mechanization interventions mainly addressing rice production areas, agroforestry Haitian farmers used to buy expensive private mechanization services^2,17^. These services mostly concern post-harvest processing (milling, transforming, packaging, etc.) and contribute to concentrating the power and the added value in this step of the value chain. When they relate to land preparation and crops wedding, their high cost and limited availability do not permit them to sow at the most appropriate time and prevent farmers from cultivating the totality of their land. Evidence-based knowledge on socioeconomic factors associated with farmers' use of mechanization can help provide adapted mechanization in the context of climate change and youth disaffection for manual farming.

## Methodology

### Study area, questionnaire, and data collection

The primary data analyzed in this research was collected in 2021 among 637 farms, selected from five geographic departments or regions (out of a total of ten in the country) targeted by the PITAG, namely the North, the North-East, the South, the Grande-Anse and the upper Artibonite. Only the North, North-East and South are partially irrigated, with rice production in the North-East, and South, and banana in the North. Maize and beans (common beans, pigeon peas and cow peas) are the most common crops cultivated in the lowlands of the five regions. Farmers in the highlands cultivate rain-fed crops like yam, banana, sweet potato, cassava, etc. Mechanical traction is rare and is provided as a paid service only in North-East and South irrigated lands, mainly for plowing. Mechanical threshing and winnowing of rice is only offered in North-East’s lowlands. Animal traction is a little more accessible for land preparation in all the regions, particularly in upper Artibonite. Farmers often use manual tools because of mostly high declivity in all the regions. Therefore, there is a growing tendency to mechanize product transformation (shelling, seeding, milling, etc.). As seedling remains manual, it is almost impossible to mechanize weeding, even in lowlands.

We selected an average of 3 municipalities from each region totaling 16. Around 40 farmers were selected from each municipality. Surveyors used a stratification strategy was based on age and gender of the farmers, farm size, production systems, and agro-ecological diversification. Data collection was done face-to-face with farmers, with printed questionnaires. The latter was designed by senior researchers who also supervised all the processes with the assistantship of young researchers.

Our sampling and sampling design is similar to Paul’s^21^ previous study in Haiti, which selected 5 regions and 15 municipalities. Similarly, we use a four-stage sampling strategy to select respondents: regions or geographic departments, municipalities, farms, and farmers.

The requested information included demography, household composition and activities, household expenses and lifestyle, different occupations, income, education, farm characteristics, agricultural activities, social interactions, access to innovations, use of mechanization, access to credit and remittances, food security, and nutrition.

### Data analysis

Based on the use of different services, we coded the use of agricultural mechanization as a binary variable with “yes” or 1 if a farmer owned, rented, or purchased any type of mechanization, and “no” or 0 otherwise. In our sample, the different types of mechanization included animal or mechanical traction, mechanical machines or tools for sowing, weeding, harvesting, winnowing, threshing, milling, etc. We also transformed the continuous variables in the dataset into scale, such as age, number of trees, income, etc. in the dataset. All variables were constructed based on previous similar studies^22–25^.

We used multivariate analysis for the typology construction^26,27^, more precisely we performed principal components analysis (PCA) for the statistical reduction of explanatory variables to homogeneous farm types, and hierarchical clustering analysis (HCA) for grouping farms into clusters. This typology construction method is a sequential and iterative process that involves four steps^23^: (i) exploratory analysis (outlier analysis, variable transformation, and correlation analysis); (ii) factor analysis and (iii) cluster analysis; (iv) assessing the reliability of clustering results. We identified the number of clusters using principal component analysis based on a selection of variables that maximized the Kaiser-Mayer-Olkin or KMO indicator (To be retained in the analysis, a variable must obtain a KMO measurement exceeding 0.5) and Bartlett's test of sphericity (p-value < 5%). Based on those criteria, twelve variables were finally retained: Agricultural mechanization, Number of irrigated plots, Level of fragmentation, Farm size, Household size, Specialization, Average number of trees, Steep slopes, External labor (Equivalent Full time) purchased, Education of farmer, Access to credit, and Remittances.

We used hierarchical cluster analysis (HCA) to assign farmers with similar characteristics to the same clusters. The HCA was performed using dendogram to illustrate how the nested clusters were cut to identify farm types. Ward aggregation method was used and the tree cut point was placed at third level. Based on these multivariate analyzes, each farmer was assigned the appropriate cluster allowing further data analysis.

We used SPSS V.20 software to perform all data analysis. Table 1 shows the definition of all the variables used in the study.

**Table 1:** Variable description.

Variables	Definition and measurement	Modalities	Frequency/mean (sd)
Mechanization use	Use of any type of agricultural mechanization, harvest and post-harvest processing mechanical tool; coded “Yes” if the farm used any type of mechanization either owned, rented, or purchased, “No” otherwise	No	39.1%
		Yes	60.9¨%
Regions	Binary variable coded “Grande-Anse”, “Artibonite”, “Nord”, “Nord-Est”, and “Sud” for the five geographic departments	Grande-Anse	32.2%
		Artibonite	30.4%
		Nord	14.5%
		Nord-Est	15.9%
		Sud	15.9%
Gender of farmer	Binary variable related to the gender of the farmer managing the farm; coded “1” for male and “0” for female	Female	14.0%
		Male	86.0%
Age of farmer	Age of the farmer coded “Young” if age is less than 45 years, “Mature” if age is between 45 and 55, and “Old” if age is higher than 55	Young	24.8%
		Mature	27.0%
		Old	48.2%
Agricultural education	Level of agricultural education of the farmer, coded “No education” if any agricultural education or training, “Seminars” if short training, “graduate education” if technical school or university agriculture	No education	68.3%
		Seminars	28.3%
		Graduate school	3.5%
Education within the farm	Average level of education among the farming household, coded “Low” if no member has a minimum of higher school, “Only one” if one member has attained higher school, and “Two or more” otherwise	Low	39.9%
		Only one	26.7%
		Two or more	33.4%
Specialization	Type of specialization of the farm members in terms of percentage of the total working time, coded “Specialized in” if most of the time is used within the farm, “Specialized out” if most of the time is used out of the farm, and “Both in and out” otherwise	Specialized in	30.6%
		Both in and out	16.8%
		Specialized out	52.6%
Farm size	The class of size of the farms coded “Very small” if less than 1 carreaux (1.29 ha), “Small” if 1–2 carreaux, and “Large” if more than 2 carreaux	Very small	58.1%
		Small	30.9%
		Large	11.0%
Livestock	The class of total livestock measured in conventional tropical livestock units (TLU) and coded “Very few” if less than 2, “Few” if 2–3.6 TLU, and “Important” if more than 3.6 TLU	Very few	36.6%
		Few	32.2%
		Important	31.2%
Irrigation	Number of irrigated plots within the farm, coded “Absence” if zero plot is irrigated, “Few” if only one plot is irrigated, and “Many” if more than one irrigated plot	Absence	82.4%
		Few	0.9%
		Many	16.6%
Adaptation to climate change	Binary variable coded “Yes” if the farming system was adapted because of climate change events suffered in the previous year, and “No” otherwise	No	63.7%
		Yes	36.3%
Farm fragmentation	Number of plots constituting the farm, coded “Low” if less than 3 plots, “Medium” if 3 plots, and “High” if more than 3 plots	Low	65.9%
		Medium	21.4%
		High	12.7%
Trees	Average number of trees within the farm	Number	82.11 (190.19)
Plots slope	Percentage of steep slopes of the farm plots	Number	11.11 (28.96)
External labor	Total external labor (equivalent full time) used by the farm yearly	Number	86.47 (118.49)
Contact with public interventions	Binary variable coded “Yes” if any member of the farm benefited a public intervention in the last five years, and “No” otherwise	No	56.5%
		Yes	43.5%
Regular saving behavior	Binary variable coded “Yes” if farm members save money regularly, and “No” otherwise	No	65.1%
		Yes	34.9%
Access to credit	Binary variable coded “Yes” if any member of the farm benefited a credit last year, and “No” otherwise	No	72.1%
		Yes	27.9%
Remittances	Binary variable coded “Yes” if any member of the farm received remittance last year, and “No” otherwise	No	49.8%
		Yes	50.2%
Off-farm income	Total amount of off-farm income (billion Haitian gourdes, HTG) earned by the farm members last year	Less than 100,000.00	18.2%
		100,000.00–200,000.00	3.9%
		More than 200,000.00	77.9%
Food security status	Food security status of the farm household based on food access during the last two months and coded into four categories according Coates et al.’s^28^ food security scale	Food secure	2.5%
		Low food insecurity	32.8%
		Moderate food insecurity	27.5%
		Severe food insecurity	37.2%

Additional statistical and econometric analyses were used to bring evidence about what factors significantly determined Haitian farmers' use of agricultural mechanization in 2021.

### The model

The model estimated in this study was a multinomial logistic regression (MNLR). It is grounded in agricultural development theory in the context of farming in developing countries which predicts that mechanization can play a critical role when high labor costs have negative effects on agricultural productivity and the welfare of smallholder farm households^5^. Following the neoclassical economic theory, in such countries, although access to capital is often limited, it is assumed that labor is abundant because of the high rate of unemployment. However, the situation has changed drastically in countries like Haiti over the past decade. Farmers have been facing high labor costs^2^ because of important migration waves^29^ and the growing non-farm economy^14^. As previously observed by Oseni and Winters^30^ in the case of Nigeria, informal economic activities like transportation (with motorcycles) and telecommunication drained the youth labor force from farms to urban areas in Haiti. Instead of benefiting farmers, rising rural wages reduce their possibility of cultivating their land. According to Pingali^31^, mechanization may become profitable as wages rise. A recent study showed that the percentage of cultivated land was always lower than the total agricultural land^17^among all size of Haitian farms. The previous official census reported that before 2010, only 7% of all Haitian farmers were equipped with mechanization tools^12^. International donors have supported governmental efforts to bring mechanization to Haitian farmers to improve their socioeconomic conditions and reduce food insecurity in the country After a decade of support, heavy mechanization remains poorly accessible, while farmers can mainly rely on small mechanization to face what was recently described by researchers as an “agricultural labor crisis” in Haiti^2^.

In our model, we consider that a farmer i (where i = 1, 2…I) earned a utility from the using mechanization. We assume that a farmer i used mechanization whenever his/her had access to it (either through ownership, rent, or purchase) and if his/her utility was superior to a threshold ẟ, whereas he/she did not if his/her utility was inferior or equal to this threshold. The Utility function ${U}_{i}^{*}$ can be explained by a deterministic part: vector ${X}_{i}$ of observable characteristics and an error term (${\varepsilon }_{i}$). For the farmer i, this utility function can be written as in the following Eq. (1):

1 $${U}_{i}^{*}=\alpha +{\beta X}_{i}+{\varepsilon }_{i}$$

The error term is independent and identically distributed, as follows: ${\varepsilon }_{i}\sim N(\text{0,1})$. The rule of decision, for each participant (farm or farmer) i is to make the choice that maximizes his/her utility function. To study the personal characteristics of the participants that explain their choice to use mechanization or not, we first define a binary variable ${y}_{i}$ that measures their choice, as follows in the Eq. (2):

2 $${P(Y}_{ij}=1)=F(m+{\beta X}_{ij}$$

In this relation, F is a cumulative density function given by the Eq. (3):

3 $$F(m+{\beta X}_{ij}={\int }_{-\infty }^{(m+{\beta X}_{ij}}\frac{1}{\sqrt{2\pi }}{e}^{-{Z}^{2}}dz.$$

The parameters m and β of the model are estimated using methods numerical maximization of the logarithm of the likelihood function which is written as follow in the Eq. (4):

4 $$ln\left[L, Y, \beta \right]={\sum }_{i=1}^{J}\left[{Y}_{i}lnln \left[F(m+{\beta X}_{ij}\right] \right]+\left(1-{Y}_{i}\right)ln\left[1-F(m+{\beta X}_{ij}\right]$$

We estimated a binary model being certain that the predictions will fall into the interval (0, 1). And, as the number of observations (637) is sufficiently high, we confidently assumed that the error term was distributed normally, what allows us to opt between a Probit and Logit model. We choose a Logit for interpretation easiness using adjusted odd ratio (AOR). The form of the equation to be estimated is then as in Eq. (5):

5 $$y_{i} = \left\{ {\begin{array}{*{20}c} {1, if U_{i}^{*} > \delta \left( {the farmer i used agricultural mechanization} \right)} \\ {0, if 0 \le \delta \left( {the farmer i did not use agricultural mechanization} \right)} \\ \end{array} } \right.$$

The variable ${Y}_{i}$ takes the value 1, if the farmer used agricultural mechanization, and 0 if he/she did not. In this case, the endogenous variable of the model is dichotomous. The linear multiple regression standard models can be written as in Eq. (6):

6 $${Y}_{i}=\alpha +{\beta X}_{i}+{\varepsilon }_{i}$$

The vector of explanatory variables ${X}_{i}$ includes characteristics related to farmers’ profile and farms’ characteristics. It also includes variables related to the external environment as they relate to farmers’ relations. Farmers’ profiles included age, gender and agricultural education. The average education of all the people on the farm was  also taken into consideration. We mobilized the existing literature^3,32,33^ to select socioeconomic variables in order to capture farming characteristics: farm size, access to credit, remittances, financial behavior, irrigation, benefit to public programs, innovation to face climate change, and food security status. The equation to be estimated for a farmer i was specified as in the Eq. 7:

7 $${\text{Mechanization Use}}_{\text{i}}= \alpha +{{\beta }_{1}Gender}_{i}+{{\beta }_{2}Age}_{i}+{{\beta }_{3}Agricultural Education}_{i}+{{\beta }_{4}Education}_{i}+{{\beta }_{5}Farm size}_{i}+{{\beta }_{6}Livestock}_{i}+{{\beta }_{7}Irrigation}_{i}+{{\beta }_{8}Adaptation to climate change}_{i}+{{\beta }_{9}Contact with Public interventions}_{i}+{{\beta }_{10}Access to credit}_{i}+{{\beta }_{11}Access to remittances}_{i}+{{\beta }_{12}Saving behavior}_{i}+{{\beta }_{13}Off Farm income}_{i}+{{\beta }_{14}Food security status}_{i}$$

After bivariate analysis using Pearson’s χ^2^ tests (p-value < 5%), we retained the following final Eq. 8 for our multinomial logistic regression (MNLR) model:

8 $${\text{Mechanization Use}}_{\text{i}}= \alpha +{{\beta }_{1}Gender}_{i}+{{\beta }_{2}Age}_{i}+{{\beta }_{3}Irrigation}_{i}+{{\beta }_{4}Adaptation to climate change}_{i}+{{\beta }_{5}Access to credit}_{i}+{{\beta }_{6}Access to remittances}_{i}+{{\beta }_{7}Saving behavior}_{i}+{{\beta }_{8}Off Farm income}_{i}+{{\beta }_{9}Food security status}_{i}$$

### Ethics approval

The survey protocol and questionnaire were approved by the technical committee of the program at the Ministry of Agriculture in the Government of Haiti led by the internal Bureau of Agricultural Statistics, an Institutional Review Board in the framework of the MARNDR/PITAG/SFQ-15/19. The participants’ anonymity and confidentiality were assured. All participants signed a Consent Agreement. All methods were carried out in accordance with relevant guidelines and regulations.

### Informed consent

Required informed consent was obtained from farmers during survey time, as it is a survey type of research.

## Results

### Haitian farmers’ use of mechanization

Our results revealed that 60.9% of all the farmers used mechanization, particularly from paid services. A significant percentage of the farmers (28.7%) owned mechanical tools, while 47.1% had to pay for services provided by mechanization micro-enterprises. This use included all kinds of mechanization services in the farm for production, harvest, and post-harvest.

The famers who have bought mechanization services have paid an average of 11,670.46 (st-dev: 1685.79) Haitian gourdes for 2021. This represented an estimated cost of more than 100 US dollars. Those who owned their mechanization equipment spent an average of 41,469.97 (st-dev: 7423.30) Haitian gourdes, representing more than 400 US dollars.

### Farmers’ typology

**Figure 1:**
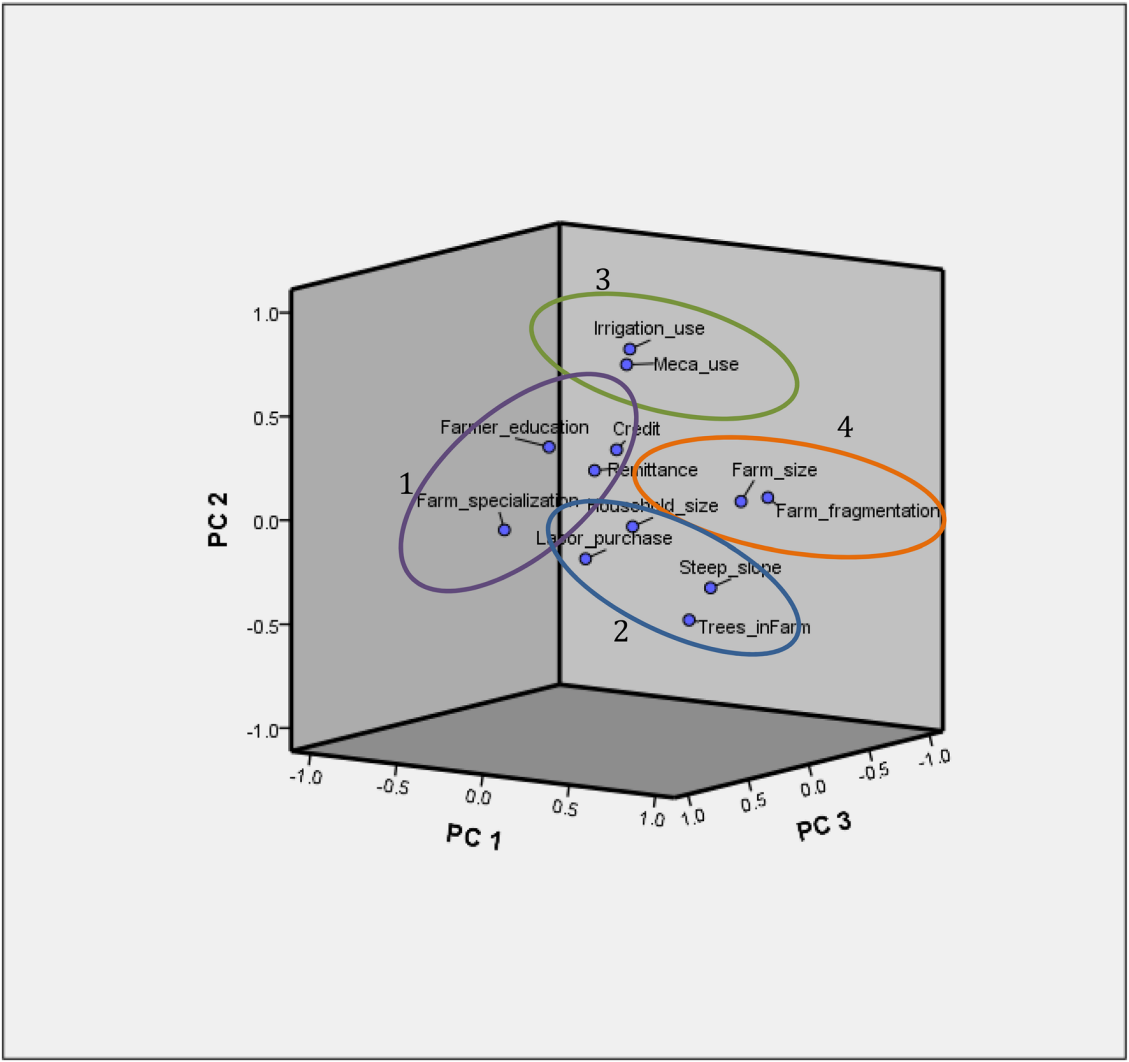
Farm types by principal components analysis. Source: The authors.

**Figure 2:**
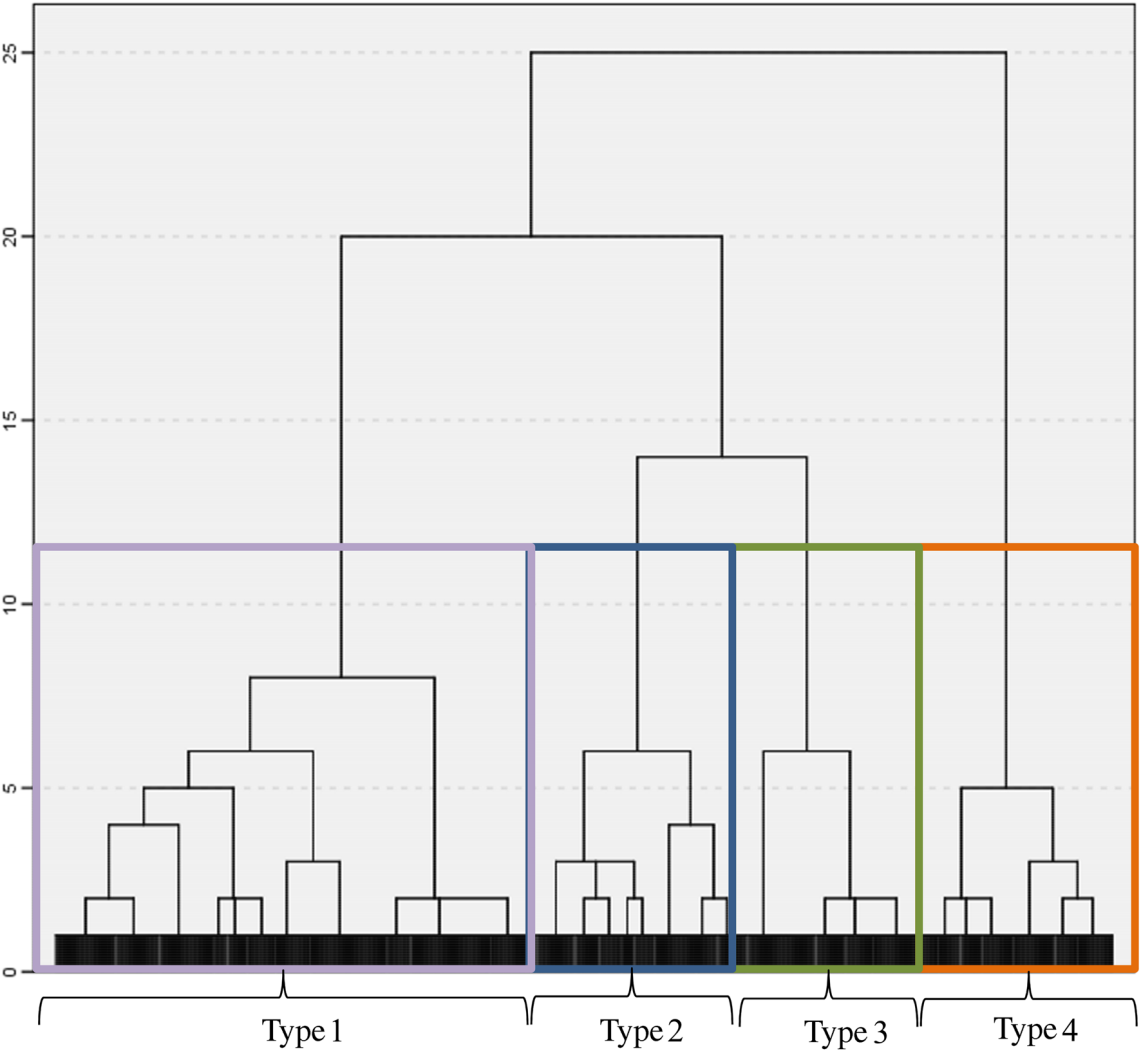
Dendogram with tree cut. Source: The authors.

Multivariate analysis conducted according the previous criteria of data analysis led to 4 different clusters. The KMO was 0.610 and Bartlett's sphericity test significance at p < 0.001. All principal components exceeding an eigenvalue of 1 were retained, and variables with contribution weight lower than 0.3 were excluded. The explained cumulative variance was ≤ 60% but with loading ≥ 0.50. As the sample was sufficiently high (637), orthogonal rotation (varimax method) was used to group study variables. Both principal component analysis (PCA) and hierarchical cluster analysis (HCA) suggested 4 categories of farmers. Figure 1 displays the three first components and Fig. 2 shows the dendogram with cut tree points.

Based on PCA’s information, the 4 clusters were named as follows: Little rain-fed farms (Cluster 2); Little lowlands farms (Cluster 2); Medium-sized farms in irrigated plains (Cluster 3), and Large fragmented mountain farms (Cluster 4). Consistently with the Haitian farming, the first cluster looks more diverse (Fig. 2).

### Farmers’ characteristics in each cluster

**Figure 3:**
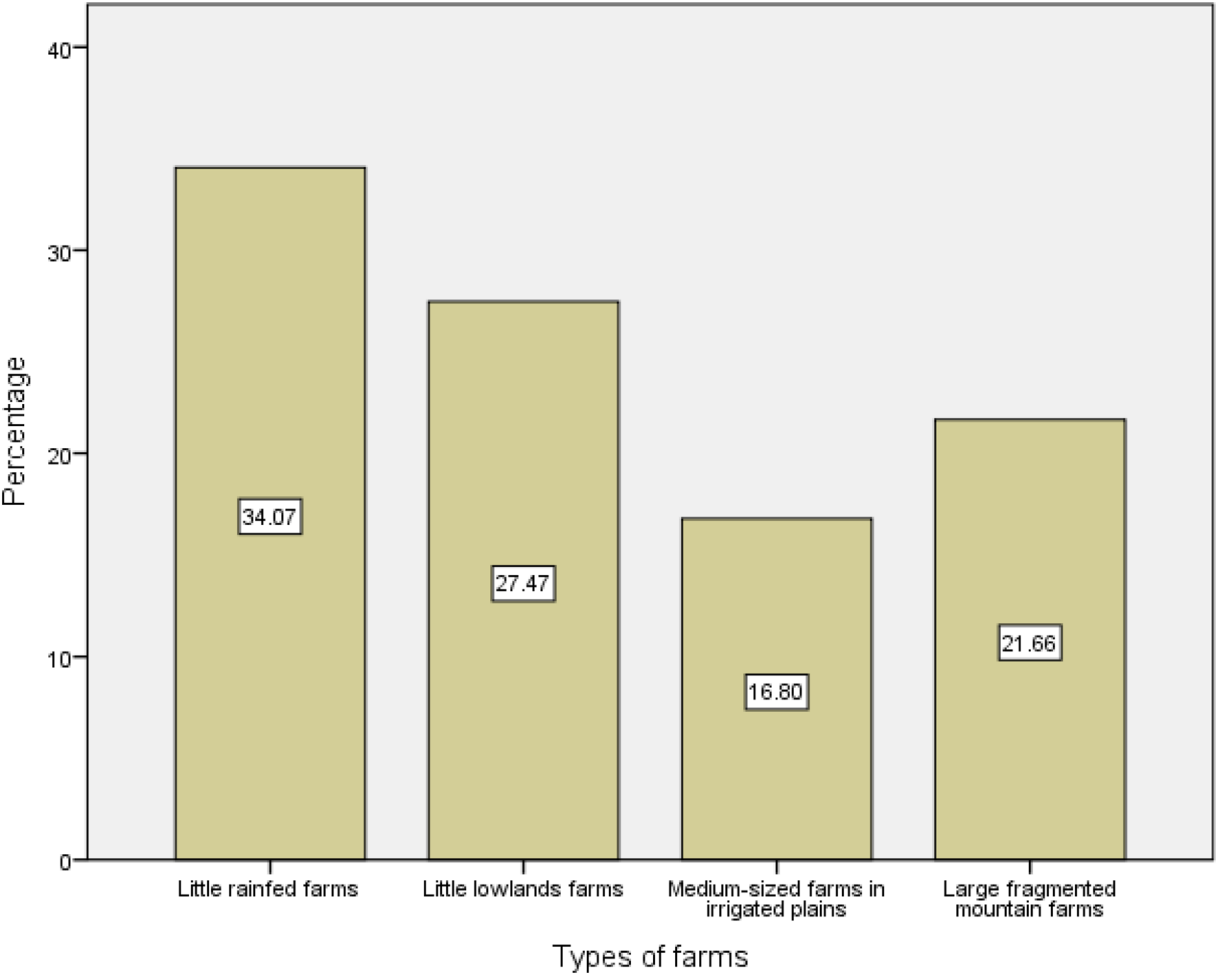
Percentage of farms in each cluster. Source: The authors.

We identified four clusters of farmers according to typology based on farmers’ and farms’ characteristics, and mechanization use (Fig. 3). Cluster 1 was the largest with 217 farmers out of the 637, and cluster 3 the smallest one in the sample with 107 farmers. The clusters 2 and 4 included 175 and 138 farmers respectively. Each cluster was unique, based on indicators presented in Table 2 below.

**Table 2:** Clusters’ selected characteristics.

Characteristics	Cluster 1	Cluster 2	Cluster 3	Cluster 4	Pooled sample
	Little rain-fed farms	Little lowlands farms	Medium-sized farms in irrigated plains	Large fragmented mountain farms	
Mechanization use	Low (47.5%)	High (60.0%)	Very high (99.1%)	Medium (53.6%)	High (60.9%)
Irrigation access	1.4%	0.0%	98.1%	2.9%	12.9%
Level of fragmentation	Low	Low	Medium	High	Low
Farm size*	0.794 (0.041)	0.663 (0.037)	1.303 (0.124)	1.842 (0.121)	1.072 (1.052)
Specialization	Mostly out of the farm	Mostly out of the farm	Fairly out of the farm	Mostly in the farm	Mostly out of the farm
Estimated average number of trees *	67.12 (7.384)	61.67 (12.102)	21.23 (4.151)	179.30 (27.181)	82.16 (190.19)
Percentage of steep slopes*	12.01 (2.336)	9.13 (2.211)	1.90 (0.965)	19.28 (2.463)	11.11 (28.96)
External labor (Equivalent Full time) *	64.90 (5.706)	50.88 (5.706)	93.86 (13.135)	158.58 (16.510)	86.47 (118.50)
Gender of farmer (% of women)	18.4%	15.4%	9.3%	8.7%	14%
Education of farmer	Mostly primary school	Mostly primary school	Mostly secondary school	Mostly primary school	Mostly primary school
Age of farmer*	54.53 (0.857)	54.45 (1.082)	50.83 (1.345)	53.15 (0.947)	53.48 (12.87)
Access to credit	27.6%	20.6%	43.9%	25.4%	27.9%
Remittances	6.9%	90.3%	59.8%	60.1%	50.2%
Off-farm income * (billion Haitian gourdes)	1.217 (0.106)	1.210 (0.128)	2.511 (0.265)	0.899 (0.083)	1.389 (1.908)
Technical or university education in agriculture	3.2%	1.1%	6.5%	4.3%	3.5%
Adapted farming system because of climate change	31.3%	41.1%	51.4%	26.1%	36.3%

*Standard deviation is given between parentheses.

Farmers in cluster 3 used more agricultural mechanization (Table 2). They were younger, more educated, more innovative in front of climate change, fairly diversified out of the farm, and working on reasonable farm size. They also had the highest access to credit, although they fairly depended on remittances. They were the farmers who earned the highest off-farm income. They were also unique in terms of irrigation access. This differentiated them from farmers in cluster 2 who cultivated rain-fed lowlands. The farmers in the first two clusters were the poorest, while farmers in the fourth cluster had larger farms located in mountainous areas.

Farmers in Little rain-fed farms (cluster 1) were the poorest ones. They were the oldest farmers, and had the lowest mechanization use. However, they were not the ones with the lowest access to credit. They had a low percentage of irrigated plots. As they were mostly specialized out of the farm, they have some off-farm income. But they were the lowest recipient of remittances, and the highest rate of women as farm managers. With farms in cluster 4, they were less likely to develop adaptation to climate change. As shown in Fig. 1, these little rain-fed farms were the most numerous in the sample.

Farmers in Little lowlands farms (cluster 2) had the second highest mechanization use. But they had no irrigated plot. Thus they were rain-fed. They were specialized out of the farm with the lowest purchased external labor. They had the lowest access to credit but the highest rate of remittances. In this cluster, farms had the lowest technical education in agriculture, but they managed to adapt themselves to climate change better than clusters 1 or 4.

The farmers cultivating medium-sized farms in irrigated plains were those grouped in cluster 3. They were the best off in almost all the categories. They had the highest level of mechanization use and the most important percentage of irrigated plots. The farm size in this cluster is the second largest. Managed by the youngest farmers in the sample, they were the more educated farmers, both formal and agricultural training, with the highest access to credit. They were privileged in terms of off-farm income, remittances reception, and development of adaptation strategies to climate change.

The farmers in cluster 4 were well-endowed in terms of natural capital (farm size). However, these “Large fragmented mountain farms” were highly fragmented, with the highest number of trees and a small percentage of irrigated plots, probably with stagnated water such as lagoon. They were the most specialized within the farms, the second lowest in terms of access to credit. They were privileged in terms of agricultural education and did not have an important need for climate change adaptation.

### Farms’ use of mechanization

Statistical analysis using χ^2^ tests (Table 3) revealed that drivers of mechanization use for farmers in cluster 1 were: region, access to credit, saving behavior and food security status. Only region and food security were significant drivers of mechanization use for farmers in cluster 2. The factors driving mechanization use in the case of farmers in cluster 3 were gender and off-farm income. Finally, farmers in cluster 4 had their mechanization use determined by region, age of the farmer, and adaptation to climate change.

**Table 3:** Relationship between farmer and farming characteristics, and use of mechanization.

Farmer and farming characteristics	Mechanization use														
	Cluster 1: Little rain-fed farms N = 217			Cluster 2: Little lowlands farms N = 175			Cluster 3: Medium-sized farms in irrigated plains N = 107			Cluster 4: Large fragmented mountain farms N = 138			Pooled sample N = 637		
	Yes N (%)	No N (%)	Sig	Yes N (%)	No N (%)	Sig	Yes N (%)	No N (%)	Sig	Yes N (%)	No N (%)	Sig	Yes N (%)	No N (%)	Sig
Regions															
Grande-Anse	23 (36.5)	40 (63.5)	***	8 (30.8)	18 (69.2)	***	1 (100.0)	0 (0.0)	ns	3 (9.4)	29 (90.6)	***	35 (28.7)	87 (71.3)	***
Artibonite	8 (50.0)	8 (50.0)		10 (47.6)	11 (52.4)		1 (100.0)	0 (0.0)		29 (69.0)	13 (31.0)		48 (60.0)	32 (40.0)	
Nord	31 (73.8)	11 (26.2)		47 (73.4)	17 (26.6)		34(100.0)	0 (0.0)		15 (75.0)	5 (25.0)		127 (79.4)	33 (20.6)	
Nord-Est	21 (30.0)	49 (70.0)		10 (47.6)	11 (52.4)		0 (0.0)	0 (0.0)		14 (63.6)	8 (36.4)		45 (39.8)	68 (60.2)	
Sud = Ref	20 (76.9)	6 (23.1)		30 (69.8)	13 (30.2)		70 (98.6)	1 (1.4)		13 (59.1)	9 (40.9)		133 (82.1)	29 (17.9)	
Gender of the farmer															
Female	14 (35.0)	26 (65.0)	*	13 (48.1)	14 (51.9)	ns	9 (90.0)	1 (10.0)	***	4 (33.3)	8 (66.7)	ns	40 (44.9)	49 (55.1)	***
Male	89 (50.3)	88 (49.7)		92 (62.2)	56 (37.8)		97 (100.0)	0 (0.0)		70 (55.6)	56 (44.4)		348 (63.5)	200 (36.5)	
Age of the farmer															
Young (< 45)	22 (46.8)	25 (53.2)	ns	25 (58.1)	18 (41.9)	ns	39 (100.0)	0 (0.0)	ns	11 (37.9)	18 (62.1)	**	97 (61.4)	61 (38.6)	*
Mature (45–45)	24 (38.1)	39 (61.9)		30 (62.5)	18 (37.5)		20 (100.0)	0 (0.0)		18 (43.9)	23 (56.1)		92 (53.5)	80 (46.5)	
Old (more than 55)	57 (53.3)	50 (46.7)		50 (59.5)	34 (40.5)		47 (97.9)	1 (2.1)		45 (66.2)	23 (33.8)		199 (64.8)	108 (35.2)	
Agricultural education															
No education = Ref	67(45.6)	80 (54.4)	ns	72 (57.6)	53 (42.4)	ns	66 (98.5)	1 (1.5)	ns	51 (53.1)	45 (46.9)	ns	256 (58.9)	179 (41.1)	ns
Seminars	34 (54.0)	29 (45.0)		31 (64.6)	17 (35.4)		33 (100.0)	0 (0.0)		19 (52.8)	17 (47.2)		117 (65.0)	63 (35.0)	
Graduate school	2 (28.6)	5 (71.4)		2 (100.0)	0 (0.0)		7 (100.0)	0 (0.0)		4 (66.7)	2 (33.3)		15 (68.2)	7 (31.8)	
Education within the farm															
Low (no one with secondary school)	34 (51.5)	32 (48.5)	ns	52 (54.7)	43 (45.3)	ns	37 (97.4)	1 (2.6)	ns	28 (50.9)	27 (49.1)	ns	151 (59.4)	103 (40.6)	ns
Only one	31 (51.7)	29 (48.3)		26 (60.5)	17 (39.5)		26 (100.0)	0 (0.0)		23 (56.1)	18 (43.9)		106 (62.4)	64 (37.6)	
Two or more	38 (41.8)	53 (58.2)		27 (73.0)	10 (27.0)		43 (100.0)	0 (0.0)		23 (54.8)	19 (45.2)		131 (61.5)	82 (38.5)	
Farm size															
Very small (< 1 cx)	74 (48.1)	80(51.9)	ns	81 (60.0)	54 (40.0)	ns	55 (98.2)	1 (1.8)	ns	10 (40.0)	15 (60.0)	ns	220 (59.5)	150 (40.5)	ns
Small (1–2 cx)	25 (43.9)	32 (56.1)		21 (56.8)	16 (43.2)		33 (100.0)	0 (0.0)		39 (55.7)	31 (44.3)		118 (59.9)	79 (40.1)	
Large (> 2 cx)	4 (66.7)	2 (33.3)		3 (100.0)	0 (0.0)		18 (100.0)	0 (0.0)		25 (58.1)	18 (41.9)		50 (71.4)	20 (28.6)	
Livestock															
Very few (< 2 UBT)	35 (42.2)	48 (57.8)	ns	38 (51.4)	36 (48.6)	ns	36 (100.0)	0 (0.0)	ns	18 (45.0)	22 (55.0)	ns	127 (54.5)	106 (45.5)	**
Few (2–3.6 UBT)	37 (56.1)	29 (43.9)		37 (67.3)	18 (32.7)		34 (97.1)	1 (2.9)		25 (51.0)	24 (49.0)		133 (64.9)	72 (35.1)	
Important (> 3.6 UBT)	31 (45.6)	37 (54.4)		30 (65.2)	16 (34.8)		36 (100.0)	0 (0.0)		31 (63.3)	18 (36.7)		128 (64.3)	71 (35.7)	
Irrigation															
Absence (0 irrigated plot)	102(47.7)	112 (52.3)	ns	70 (40.0)	105 (60.0)	ns	2 (100.0)	0 (0.0)	ns	71 (53.0)	63 (47.0)	ns	280 (53.3)	245 (46.7)	***
Few (1 irrigated plot)	0	0		0	0		3 (100.0)	0 (0.0)		1 (66.7)	1 (33.3)		5 (83.3)	1 (16.7)	
Many (> 1 irrigated plot)	1 (33.3)	2 (66.7)		0	0		101 (99.0)	1 (1.0)		1 (100.0)	0 (0.0)		103 (97.2)	3 (2.8)	
Adaptation to climate change															
No = Ref	67 (45.0)	82 (55.0)	ns	56 (54.4)	45 (45.6)	*	51 (98.1)	1 (1.9)	ns	49 (48.0)	53 (52.0)	**	223 (54.9)	183 (45.1)	***
Yes	36 (52.9)	32 (47.1)		49 (68.1)	23 (31.9)		55 (100.0)	0 (0.0)		25 (69.4)	11 (30.6)		165 (71.4)	65 (28.6)	
Contact with public interventions															
No = Ref	58 (46.8)	66 (53.2)	ns	67 (61.5)	42 (38.5)	ns	65 (100.0)	0 (0.0)	ns	35 (56.5)	27 (43.5)	ns	225 (62.5)	135 (37.5)	ns
Yes	45 (48.4)	48 (51.6)		38 (57.6)	28 (42.4)		41 (97.6)	1 (2.4)		39 (51.3)	37 (48.7)		163 (58.8)	114 (41.2)	
Access to credit															
No = Ref	67 (42.7)	90 (57.3)	**	82 (59.0)	57 (41.0)	ns	59(98.3)	1 (1.7)	ns	52 (50.5)	51 (49.5)	ns	260 (56.6)	199 (43.4)	***
Yes	36 (60.0)	24 (40.0)		23 (63.9)	13 (36.1)		47 (100.0)	0 (0.0)		22 (62.9)	13 (37.1)		128 (71.9)	50 (28.1)	
Remittances															
No = Ref	95 (47.0)	107 (53.0)	ns	7 (41.2)	10 (58.8)	*	42 (97.7)	1 (2.3)	ns	33 (60.0)	22 (40.0)	ns	177 (55.8)	140 (44.2)	***
Yes	8 (53.3)	7 (46.7)		98 (62.0)	60 (38.0)		64 (100.0)	0 (0.0)		41 (49.4)	42 (50.6)		211 (65.9)	109 (34.1)	
Regular saving behavior															
No = Ref	65 (41.7)	91 (58.3)	***	56 (56.0)	44 (44.0)	ns	72 (98.6)	1 (1.4)	ns	50 (58.1)	36 (41.9)	ns	243 (58.6)	172 (41.4)	*
Yes	38 (62.3)	23 (37.7)		49 (65.3)	26 (34.7)		34 (100.0)	0 (0.0)		24 (46.2)	28 (53.8)		145 (65.3)	77 (34.7)	
Off-farm income															
Less than 100,000.00 HTG = Ref	22 (47.8)	24 (52.2)	ns	15 (50.0)	15 (50.0)	ns	13 (92.9)	1 (7.1)	**	14 (53.8)	12 (46.2)	ns	64 (55.2)	14 (44.8)	*
100,000.00–200,000.00 HTG	3 (50.0)	3 (50.0)		2 (40.0)	3 (60.0)		3 (100.0)	0 (0.0)		3 (27.3)	8 (72.7)		11 (44.0)	14 (56.0)	
More than 200,000.00 HTG	78 (47.3)	87 (52.7)		88 (62.9)	52 (37.1)		90 (100.0)	0 (0.0)		57 (56.4)	44 (43.6)		313 (63.1)	183 (36.9)	
Food security status															
Food secure	3 (27.3)	8 (72.7)	***	100 (100.0)	0 (0.0)	**	4 (100.0)	0 (0.0)	ns	0	0	ns	8 (50.0)	8 (50.0)	***
Low food insecurity	33 (66.0)	17 (34.0)		49 (72.1)	19 (27.9)		70 (100.0)	0 (0.0)		11 (52.4)	10 (47.6)		163 (78.0)	46 (22.0)	
Moderate food insecurity	34 (51.5)	32 (48.5)		26 (49.1)	27 (50.9)		18(94.7)	1 (5.3)		15 (40.5)	22 (59.5)		93 (53.1)	82 (46.9)	
Severe food insecurity = Ref	33 (36.7)	57 (63.3)		29 (54.7)	24 (45.3)		14 (100.0)	0 (0.0)		48 (60.0)	32 (40.0)		124(52.3)	113 (47.7)	
Total	103 (47.5)	114 (52.5)		105 (60.0)	70 (40.0)		106 (99.1)	1 (0.9)		74 (53.6)	64 (46.4)		388 (60.9)	249 (39.1)	

Significance threshold: ***significant at 1%; ** significant at 5%; and * significant at 10%. In addition, ns means “non significant”.

In the pooled sample, the factors significantly linked to mechanization use were: region, gender of the farmer, access to irrigation, adaptation to climate change, livestock credit and remittances and food security status (Table 3). Factors like contact with public interventions (namely agricultural extension), education and agricultural training, farm size, and livestock were not significant determinants of mechanization use.

We estimated the model (8) with logistic regression for the four clusters separately and the pooled sample. The non-significant variables in all the categories of Table 3 were discarded. As there was no significant variation between the farms inside cluster 3, we merged clusters 2 and 3 (both were in the lowlands, but cluster 2 was rain-fed while cluster 3 was irrigated) for a better model fit.

The tests on the quality of the models showed that they were globally significant. Their explanatory power expressed by the pseudo R squared of Cox & Snell and Nagelkerke allows us to consider they were useful to study the farms’ agricultural mechanization. The test for multicollinearity revealed that all the models had acceptable variance inflation factor (VIF). All VIFs ranged between 1.019 and 1.135 for the variables, and the mean VIF was less than 1.210 for all the models. The determinants of the use of agricultural mechanization are presented in the following Table 4.

**Table 4:** Drivers of farmers mechanization use (multinomial logistic regression model).

Farmer and farming characteristics	Cluster 1: Little rain-fed farms		Clusters 2 and 3: Lowlands farms		Cluster 4: Large fragmented mountain farms		Pooled sample	
	Estimates	Adjusted odds ratio (AOR)	Estimates	Adjusted odds ratio (AOR)	Estimates	Adjusted odds ratio (AOR)	Estimates	Adjusted odds ratio (AOR)
Regions								
Grande-Anse	− 2.416	0.089***	− 2416	0.089***	− 3.112	0.045***	− 2.110	0.121***
Artibonite	− 1.376	0.253*	− 0.913	0.401	1.123	3.073	− 0.002	0.998
Nord	− 0.548	0.578	− 0.075	0.928	1.760	5.813*	0.167	1.182
Nord− Est	− 2.041	0.130***	− 1.657	0.191***	0.122	1.130	− 1.289	0.276***
Sud = Ref								
Gender of the farmer								
Female = Ref								
Male	− 0.224	0.779	− 0.349	0.706	− 0.95	0.387	− 0.230	0.795
Age of the farmer								
Young (less than 45) = Ref								
Mature (45–45)	− 0.484	0.616	0.179	1.196	0.208	1.231	0.047	1.048
Old (more than 55)	0.742	2.099*	0.224	1.251	0.896	2.449	0.569	1.776**
Livestock								
Very few (< 2 UBT)								
Few (2–3.6 UBT)							0.551	1.736**
Important (> 3.6 UBT)							0.508	1.662*
Irrigation								
Absence (0 irrigated plot) = Ref								
Few (1 irrigated plot)							1.228	3.379
Many (> 1 irrigated plot)							2.508	12.274***
Adaptation to climate change								
No = Ref								
Yes	− 0.170	0.844	0.542	1.719	0.984	2.675*	0.195	1.215
Access to credit								
No = Ref								
Yes	1.175	3.240***	1.102	3.010**	1.339	3.816**	0.994	2.701***
Remittances								
No = Ref								
Yes	− 0.192	0.825	− 0.346	0.708	− 0.371	0.69	− 0.002	0.998
Regular saving behavior								
No = Ref								
Yes	1.139	3.124***	0.095	1.100	− 1.014	0.363*	0.215	1.371
Off-farm income								
Less than 100,000.00 HTG = Ref								
100,000.00–200,000.00 HTG	0.120	1.127	0.525	1.691	− 0.189	0.828	− 0.124	0.884
More than 200,000.00 HTG	0.028	1.029	0.657	1.93	1.674	5.333***	0.490	1.633**
Food security status								
Food secure	− 0.362	0.696	19.972	4.71E+08			0.036	1.037
Low food insecurity	1.271	3.564**	1.029	2.797**	0.848	2.334	1.028	2.795***
Moderate food insecurity	0.333	1.385	0.073	1.075	0.855	2.351	0.310	1.363
Severe food insecurity = Ref								
Total (N)	217		282		138		637	
Log likelihood	65.663***		71.953**		63.781***		229.524***	
Pseudo R-squared	0.348		0.333		0.494		0.41	

***Significant at 1%; **significant at 5%; and * significant at 10%.

The results showed that agricultural mechanization use among “Little rain-fed farms” (cluster 1) was significantly and positively determined by three factors: regions or department of location, access to credit, regular saving behavior, and low food security status. For the “Little lowlands farms” (cluster 2) and the “Medium-sized farms in irrigated plains” (cluster 3), the mechanization use was determined by regions, access to credit, and low food security status. Location, access to credit, and high off-farm income were the significant determinants for farm’s mechanization use in cluster 4.

The results also revealed that in the pooled sample, the significant drivers of mechanization use were: regions, age of the farmers, livestock ownership, irrigation access, credit access, and low food insecurity.

## Discussion

An important percentage of the Haitian farms (60.9%) used some kind of agricultural mechanization in Haiti in 2021. This result brings evidence about the growing market for agricultural mechanization in Haiti, as labor shortage remains a critical and increasing issue for Haitian farmers^2,34^. It confirms Daméus and Jules’^2^ recent findings on profitable business opportunities for private mechanization service providers. Farms with high mechanization use were located in Sud and Nord with more irrigation access. This result confirms previous study according to which agricultural mechanization has long been limited to Haitian farmers who cultivate irrigated lowland^17^, despite most of the agricultural production used in the local food systems is realized in highlands where rain-fed crops prevail^2^. Public interventions in agricultural mechanization have mainly focused on irrigated plains which are very limited^20^. Our results also revealed that small mechanization using animal traction (26.2%) predominated over the use of tractors (9.6%) in Haiti.

However, this mechanization use varied largely between clusters of farmers. According to the farmer typology, 47.5% of “Little rain-fed farms” (cluster 1), 60.0% of “Little lowlands farms” (cluster 2), 99.1% of “Medium-sized farms in irrigated plains” (cluster 3), and 53.6% of “Large fragmented mountain farms” (cluster 4) used agricultural mechanization in 2021. Farmers in the largest cluster (cluster 1) had the lowest use of mechanization, while those in the smallest cluster (cluster 3) had the highest use. This result is consistent with the previous studies highlighting the limited mechanization of Haitian agriculture^2,12^.

In “Little rain-fed farms” (cluster 1), mechanization was significantly associated with socioeconomic factors such as location, access to credit, regular saving behavior, and low food security status. Mechanization among farms located in lowlands, including “Little lowlands farms” (cluster 2) and “Medium-sized farms in irrigated plains” (cluster 3), was significantly associated with economic factors such as credit and low food security status. This result is quite intuitive because lowland farms are often more profitable in Haiti; and private credit programs mainly target such farmers, who are more likely to reimburse their loans than their counterparts in less profitable areas. In addition, farmers in lowlands are more likely to produce cash crops, which can help reimburse agricultural loans^35^. Location, access to credit, and high off-farm income were the significant drivers of farm mechanization in “Large fragmented mountain farms” cluster 4. The latest result can be explained by the specialization primarily within the farms of farmers in the highlands, and as they naturally need post-harvest mechanization (tree crops) which is not supported by public intervention, farmers who earn high off-farm income were 5.33 odds more likely to use mechanization. Access to credit was the first and most influential driver of farm mechanization among all the types of Haitian farmers. This result is consistent with previous studies^36,37^. The second common driver was the location which reflects the agro-ecological zone, consistent with previous studies^38,39^.

This study contributed to identifying new drivers of agricultural mechanization and is in line with previous studies supporting that the successful application of agricultural mechanization requires a strong, target-oriented approach^40^. In the most mechanized farms (cluster 3) and the pooled sample, we have found that the farmers in Grande-Anse or Nord-Est were less likely to use mechanization than their counterparts in Sud. This result can be explained by the presence of services and equipment providers in this region, such as Ateliers Ecole de Camp Perrin (AECP) and the Organization for the Rehabilitation of the Environment (ORE). The pre-independence irrigation infrastructure, namely Canal d’Avezac built in 1759, can also explain why this region is better off. Farmer’s age and gender were not significant drivers of agricultural mechanization in any cluster. This result is in contrast with the literature^36–38,41^. One possible explanation is the fact that in Haiti, mechanized crops are more often managed by male farmers aged more than 45, while women are mostly involved in post-harvest processing^42^, and male farmers may ignore post-harvest mechanization. Another explanation can be found in the financial power held by women called Madan Sara, who have enough financial resources to buy agricultural mechanization services but are involved in commercialization rather than production^43^. Another important driver of mechanization use among all farmers was access to credit. Additional data analysis showed that 95.5% (p-value < 5%) of the farmers with both mechanization and credit access were using the credit to buy mechanization services. This result is consistent with Ghosh’s^32^ study of mechanization in West Bengal.

Off-farm income was a significant driver of mechanization use in the pooled sample and distinctively in cluster 4. In the poor context of Haiti, the primary internal source of income to finance agricultural mechanization may come from non-agricultural activities. This result is consistent with Gebiso et al.^38^, but contrasts with Mukherjee^37^. Among the farmers, being in an acceptable food security status (an indicator of good socioeconomic condition) was a significant driver of mechanization for all the farms, except for those in cluster 4. Farmers with low food security status had 2.79 times (AOR 2.795; 95% CI 1.588–4.922) higher odd to use mechanization than their counterparts with severe food security status. This result adds to the existing literature on mechanization determinants.

The mechanization use among the farmers cultivating “Little rain-fed farms” was also determined by regular saving behavior. This result is relatively new and intuitive. As mechanization is not for free, the farms that used to save money regurlayhad more than 3 times the odds of having mechanization access. Gender and age of the farmers were not significant determinants of mechanization use in any separate cluster. These results are in contrast with Ghosh^32^.

Remittance was not a significant driver of mechanization use in any type of studied farmers. This result is consistent with previous observations made by Innazent et al.^24^ and Ellis^44^. One possible explanation was that remittance may be mostly used for food rather than to finance agricultural mechanization by smallholder Haitian farmers.

Livestock and irrigation were significant drivers of agricultural mechanization only for farmers in the pooled sample. The particular role of irrigation in predicting mechanization use is consistent with Ghosh^32^ and Arun et al.^3^. Previous study Arun et al.^3^ found that livestock’s presence partly useful for animal traction was an important driver of mechanization. In our sample, irrigation was the most powerful driver. The farmers cultivating irrigated plots were more than 12 times (AOR 12.274; 95% CI 3.643–41.361) more likely to use mechanization than their counterparts in rain-fed lands. However, irrigation was not a discriminant factor between clusters.

Our results partly disagree with those of Arun et al.^3^ and Ghosh^32^ who found that farm size was a significant determinant variable of total investment in farm mechanization.. One possible explanation is that the natural capital (farm size, livestock) was not oriented to agricultural mechanization in the context of poor farming in Haiti.

### Strengths and limitations

This study is, to our knowledge, the first to propose a farm’s typology based on agricultural mechanization in Haiti. It contributes to the literature by testing new drivers of agricultural mechanization such as food security status, and off-farm income.

Some limitations need to be considered. First, we used cross-sectional data collected in half of the Haitian territory. The results may not be generalized. Although the pooled sample had a significant size, the model estimate on clusters used limited sub-sample size.

## Conclusion

In this article, we aimed to propose a farm typology related to mechanization use among Haitian farmers. We use a relatively large and stratified sample of 637 farms to calculate the average access and develop a farm typology based on this use. We also estimated a binary logit model that helped reveal the significant drivers of agricultural mechanization in each cluster and the pooled sample.

According to the results, more than 3 out of 5 Haitian farms (60.9%) used agricultural mechanization in 2021, mostly from the market. The studied farms were divided into four different clusters, namely “Little rain-fed farms”, “Little lowlands farms”, “Medium-sized farms in irrigated plains”, and “Large fragmented mountain farms”. The “Little lowlands farms” and the “Medium-sized farms in irrigated plains” were those with more important use of agricultural mechanization.

The overall use of agricultural mechanization was significantly influenced by the following factors: regions or geographic location, irrigation, access to credit, adaptation to climate change, off-farm income and low food security status. More precisely, the drivers of agricultural mechanization were regions, access to credit, saving habit, and low food security status in cluster 1; regions, access to credit and food security status in clusters 2 and 3 together; and regions, access to credit and off-farm income in cluster 4. They represent factors that donors and government should take into account while designing targeted agricultural mechanization programs. Such programs should remain sensible to the higher sustainability of small and appropriate mechanization as suggested by Takeshima et al.^5^, particularly in countries like Haiti where the great majority of the farms are small-scale ones in highlands or mountainous areas. Improving access to credit will also play a key role in the agricultural mechanization of any type of farmers studied.

The findings uncover the existence of an emerging market for agricultural mechanization in the context of small-scale farming mostly unable to own individual agricultural machines. Appropriate technologies—like small mechanization or intermediate machinery—are needed, with special attention to the farms in clusters 1 and 4 which are mostly adapted for agro-ecology, fruits and trees. A small mechanization project, in the framework of the PITAG, is co-designing with farmers small and low-cost machines for different farming activities (plowing, sowing, weeding, harvesting, and grain processing) that can help them reduce the hardship of agricultural activities and make them more productive without damaging the environment. This strategy can be paired with the extension of animal traction to reduce dependence toward volatile price of fossil fuel-based energy. Actors like women, also called Madan Sara, can be good partners for selling agricultural mechanization to farmers, but their market power needs to be monitored.

## Data Availability

The data presented in this study are available on reasonable request from the corresponding author.
